# A Passive Microfluidic Device Based on Crossflow Filtration for Cell Separation Measurements: A Spectrophotometric Characterization

**DOI:** 10.3390/bios8040125

**Published:** 2018-12-09

**Authors:** Vera Faustino, Susana O. Catarino, Diana Pinho, Rui A. Lima, Graça Minas

**Affiliations:** 1 Microelectromechanical Systems Research Unit (CMEMS-UMinho), University of Minho, Campus de Azurém, 4800-058 Guimarães, Portugal; id5778@alunos.uminho.pt (V.F.); scatarino@dei.uminho.pt (S.O.C.); 2 MEtRICs, Mechanical Engineering Department, University of Minho, 4800-058 Guimarães, Portugal; diana@ipb.pt; 3 Research Centre in Digitalization and Intelligent Robotics (CeDRI), Instituto Politécnico de Bragança, Campus de Santa Apolónia, 5300-253 Bragança, Portugal; 4 CEFT, Faculdade de Engenharia da Universidade do Porto (FEUP) Rua Roberto Frias, 4200-465 Porto, Portugal

**Keywords:** microfluidics, red blood cells, separation, cross-flow filtration, spectrophotometry

## Abstract

Microfluidic devices have been widely used as a valuable research tool for diagnostic applications. Particularly, they have been related to the successful detection of different diseases and conditions by assessing the mechanical properties of red blood cells (RBCs). Detecting deformability changes in the cells and being able to separate those cells may be a key factor in assuring the success of detection of some blood diseases with diagnostic devices. To detect and separate the chemically modified RBCs (mimicking disease-infected RBCs) from healthy RBCs, the present work proposes a microfluidic device comprising a sequence of pillars with different gaps and nine different outlets used to evaluate the efficiency of the device by measuring the optical absorption of the collected samples. This latter measurement technique was tested to distinguish between healthy RBCs and RBCs chemically modified with glutaraldehyde. The present study indicates that it was possible to detect a slight differences between the samples using an optical absorption spectrophotometric setup. Hence, the proposed microfluidic device has the potential to perform in one single step a partial passive separation of RBCs based on their deformability.

## 1. Introduction

Microfluidic devices have been widely reported to perform blood separation experiments [1,2,3,4,5]. Some diseases, such as malaria, diabetes mellitus, and sickle cell disease influence the red blood cells’ (RBCs) stiffness and, consequently, their deformability [6,7,8,9,10,11,12,13,14,15,16]. However, the complexity and peculiar characteristics of blood make it a very complicated and interesting fluid to study. It has been shown that blood behaves as a single phase, homogeneous fluid or a multiphase, non-homogeneous fluid [17,18].

Malaria is a parasitic disease with more than half of the world’s population at risk that causes around 500 thousand deaths per year, with 80% of infections occurring in children under five years old. The control, effective treatment, and elimination of this disease require an early and accurate diagnosis. The malaria parasite lifecycle passes from the mosquito vector to the human host by entering the liver cells where it matures, is released into the blood stream, and invades the RBCs. At this stage, the infected RBCs suffer biochemical, optical, and morphological changes [19,20], making these cells thicker and more rigid, resulting in a decrease of the cells’ deformability [21]. Hemodynamic studies help to obtain information regarding the presence, stage, and evolution of the disease. Particularly, the RBCs’ deformability can work as relevant biomarkers for malaria diagnostic applications [15], since they are directly related to the changes that the parasite causes throughout the evolution of the disease [22].

The separation of the blood components from the plasma could give us tools to discover new biomarkers and new ways to analyze the blood components (RBCs, white blood cells (WBCs) or even particles) separately by using one single microdevice. However, blood is a complex fluid that involves careful preparation for in vitro studies to overcome several blood flow challenges that happen within the microchannels, such as coagulation and sedimentation [23]. For example, Chen et al. [16] used cross-flow pillars in their microchannels to avoid cell clogging and jamming, and at the same time to create the cross-flow effect and multilevel filtration barriers that simultaneously separate the WBCs, RBCs, and the plasma [16]. Other authors have used microchannels with hyperbolic shape contractions to measure RBC deformability in both physiological and pathological situations [8,16,24,25,26,27,28,29,30,31,32] and they have used low concentrations to improve their measurements. Recently, Pinho et al. [25] have proposed a continuous microfluidic device for the partial separation of RBCs and the subsequent measurement of their deformability in one single device. The geometries used in this device were slightly bigger than the size of the cells and as a result this methodology was able to generate mechanical stimuli close to in vivo capillaries.

The device implemented by Pinho et al. [25] follows the work published by Rodrigues et al. [31], where the authors developed a microfluidic device with pillars to separate and collect RBCs based on their deformability, with the expectation in the future to use this device with real malaria effects in RBCs. To validate and quantify the separation of the cells based on their deformability, the present work proposes the use of an optical absorption spectrophotometric setup to compare the optical absorption of the healthy RBCs (as studied in [33]) with the optical absorption of glutaraldehyde chemically modified RBCs. By obtaining different absorption spectra for the samples according to the rate of the healthy/glutaraldehyde-induced rigid RBCs (mimicking the malaria effects) that were collected in each Eppendorf tube. Hence, this study aims to show the potential for a cross-flow microfluidic device with pillars, not only to perform the partial separation of RBCs, but also to deform cells and assess their deformability. To the best of our knowledge, there is no work in the literature quantifying RBC separation using spectrophotometric approaches (and, thus, differentiating between healthy and glutaraldehyde-induced rigid RBCs), showing the innovation of the proposed methodology. 

## 2. Experimental Procedure

This section presents the geometry, experimental setup, and materials used in the procedures for evaluating RBC separation.

### 2.1. Microchannel Geometry and Experimental Set-Up

**Figure 1:**
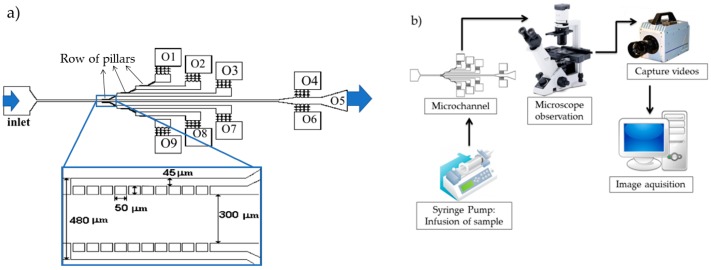
(**a**) Microchannel geometry with nine outlets (O1–O9). The arrows indicate the positioning of the rows of pillars in the microfluidic device. The left arrow indicates the region where the pillars have a 17 µm spacing between them, the central arrow indicates the region where the pillars have a 16 µm spacing between them, and the right arrow indicates the region where the pillars have a 14 µm spacing between them. The zoomed area beneath the microfluidic device represents two rows of pillars where each pillar has a 50 × 50 µm dimension and is separated from its neighbors by 17 µm; (**b**) Experimental setup comprising a high-speed camera, inverted microscope, and syringe pump system.

A polydimethylsiloxane (PDMS) (Sylgard^®^ 184 Silicone Elastomer, from Dow Corning, Midland, Michigan) microchannel with 3 × 2 rows of 10 pillars (with distances between them of 17 µm, 16 µm, and 14 µm, respectively) and a depth of approximately 30 µm was used in this study, as shown in Figure 1a). The microchannel was fabricated in the CMEMS Research Unit at the University of Minho, using soft lithography techniques with SU-8 molds [34]. The rows of pillars were placed in different levels on the microchannel to create different levels of separation. In this work, three different sets of pillars were considered (leading to the O1, O2, O3, O7, O8, and O9 outlets with hyperbolic contractions), and three additional outlets represented fluid paths with no pillars (although O4 and O6 also have hyperbolic contractions to allow deformability assessments). 

The high-speed video microscopy system used in the present study consisted of an inverted microscope (IX71, Olympus, Tokyo, Japan) combined with a high-speed camera (Fastcam SA3, Photron, CA, USA), as shown in Figure 1b. The PDMS microchannel was placed and fixed in the microscope and the flow rate of the working fluids was kept constant at 50 µL/min using a syringe pump (NEMESYS) with a 5 mL syringe. At the same time, the images of the flowing cells at the established flow rate were captured by the high-speed camera at a frame rate of 2000 frames/s and a shutter speed ratio of 1/75,000, which minimized the dragging of the cells at the high-flow rate in study. All the experimental assays were performed at room temperature (T = 22 ± 1 °C).

### 2.2. Working Fluids

Human blood from a healthy donor was collected into 2.7 mL tubes (S-Monovette^®^, Sarstedt, Germany) containing ethylenediaminetetraacetic acid (EDTA). The RBCs were taken from a female volunteer. All procedures for the collection of blood and in vitro blood experiments were carried out in compliance with the EU directives 2004/23/CE and 2006/17/CE. The whole blood was centrifuged at 1500 rpm for 15 min at 20 °C. The plasma and the buffy coat were removed and the RBCs were re-suspended and washed once in physiological salt solution (PSS) with 0.9% NaCL (B. Braun Medical, Melsungen, Germany). A RBC solution of 1% of Hematocrit (Hct) with 0.02% of glutaraldehyde was prepared in PSS and incubated for 10 min. The working fluid used was Dex40 (Sigma-Aldrich, St. Louis, MO, USA) solution containing 1% of healthy RBCs and 1% of RBCs incubated with glutaraldehyde (from now on, this solution will be referred to as the initial solution). It was used with a low Hct compared with the physiological because by using this low concentration of cells, it was possible to perform better visualizations of the flowing RBCs and consequently to obtain more accurate measurements. Each outlet of the microchannel device was connected to an Eppendorf tube to collect the samples. Once separation within the microfluidic device was finished, and in order to assure equal conditions for each optical assay, the same volume was collected from each Eppendorf tube (50 µL) and diluted with Dex40 (3 mL) to be analyzed in the spectrophotometry equipment.

### 2.3. Spectrophotometric Set-Up and Data Analysis

To quantify the separation efficiency, a spectrophotometric set-up was used that consisted of an Oriel/Newport 68,931 power supply, a model 487 picoammeter/voltage source (Keithley Instruments, Cleveland, OH, USA), an ultraviolet (UV) light source, an Oriel Newport (model 74125) monochromator, an optical fiber, and a photodiode. The photodiode converts into a currentthe light that passes through a sample placed inside a 1 cm optical path quartz cuvette. This photodiode current measured by the picoammeter was then exported to a computer using a data acquisition application developed in the LabView software. The transmittance (*T* = *I*/*I_0_*) was then used to calculate the optical absorbance: *A* = −log10(*I*/*I_0_*), where A is the absorbance, *I* the intensity of light transmitted through the sample, and *I_0_* the intensity of the incident light in the sample.

## 3. Results and Discussion

**Figure 2:**
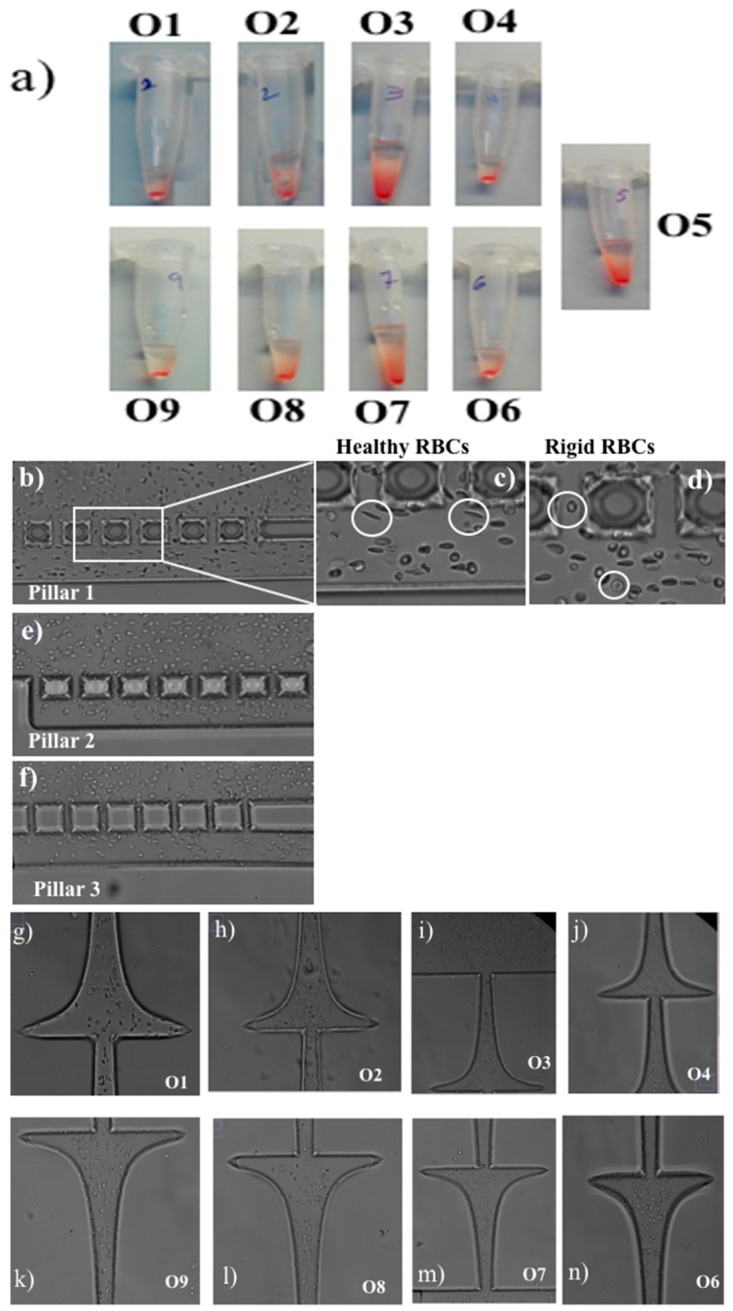
(**a**) Photo of the Eppendorf tubes with the samples collected in each outlet of the device; (**b**) pillars with a spacing of 17 µm; (**c**) elongated RBC; (**d**) rigid RBC; (**e**,**f**) pillars with spacing of 16 µm and 14 µm, respectively; (**g**–**n**) RBCs in each outlet O1–O9 of the microchannel (except for O5, where there was no hyperbolic contraction).

The proposed device was primarily evaluated by comparing the shape of the RBCs and the volume of samples collected in each outlet of the device. Figure 2 presents an overview of the samples collected in each outlet of the microfluidic device (Figure 2a), as well as microscope images of RBCs in each of those outlets and pillars.

From Figure 2 it can be observed that the separation device was able to transport the RBCs through the microfluidic channels and pillars according to their deformability. Figure 2b–f present instantaneous frames of RBCs crossing the three rows of pillars of the microfluidic device. Additionally, Figure 2c,d (magnified figures of Figure 2b) show, respectively, deformable RBCs and rigid RBCs flowing within the first row of pillars (cells located inside the white circles). By analyzing these figures, it is clear that the healthy RBCs have a high elongation when subjected to high shear flow (Figure 2c), and as a consequence it is reflected in the high deformability of these cells. On the other hand, in Figure 2d, we can see that the RBCs kept their almost spherical shape, indicating their difficulty to deform, which is a result of their glutaraldehyde-induced rigidity. These qualitative results indicate that the proposed device has the potential to measure the RBCs’ deformation index within the pillars and at the hyperbolic contractions located at the outlets, as displayed in Figure 2g–n. Additional and detailed measurements of RBCs’ deformability under hyperbolic contractions can be found in [8,35]. Figure 2a shows the Eppendorf volume of each outlet collected during the experiment.

**Figure 3:**
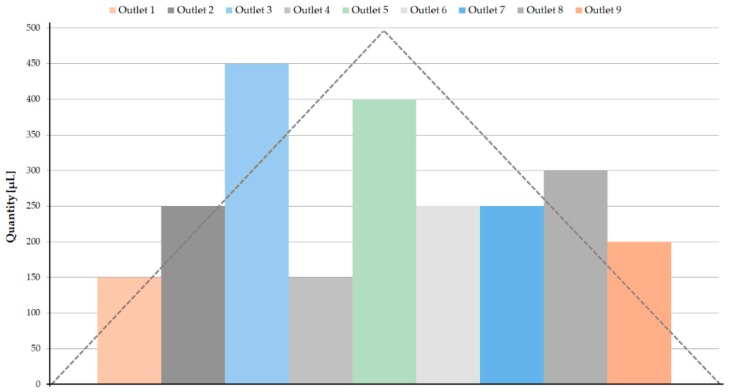
Volumes of the samples collected in the Eppendorf tubes. The dashed line represents an approximation of the expected behavior of the collected sample volumes in each outlet.

The Eppendorf volumes present some differences between the symmetric outlets (O1–O9; O2–O8; O3–O7; O4–O6), as shown in Figure 3. It was expected that the central outlets (considering O5 as the central outlet, as in Figure 3) would have higher collected volumes, and that these volumes would gradually decrease towards O1 and O9. On the upper side of the device (see Figure 1a), O1, O2, and O3 were the outlets following the 14 µm, 16 µm, and 17 µm pillar paths and, as expected, as the spacing between the pillars increased, the collected volume also increased. It was expected that the symmetric outlets would obtain similar volumes in both Eppendorf tubes, however, this phenomenon was not observed. This may indicate that the flow inside the microchannel was not fully steady. Regarding the central outlets (O4, O5, and O6), since there were no pillars in their way, the RBCs followed the easiest path, avoiding the hyperbolic contractions of O4 and O6, and explaining the higher volume of O5.

**Figure 4:**
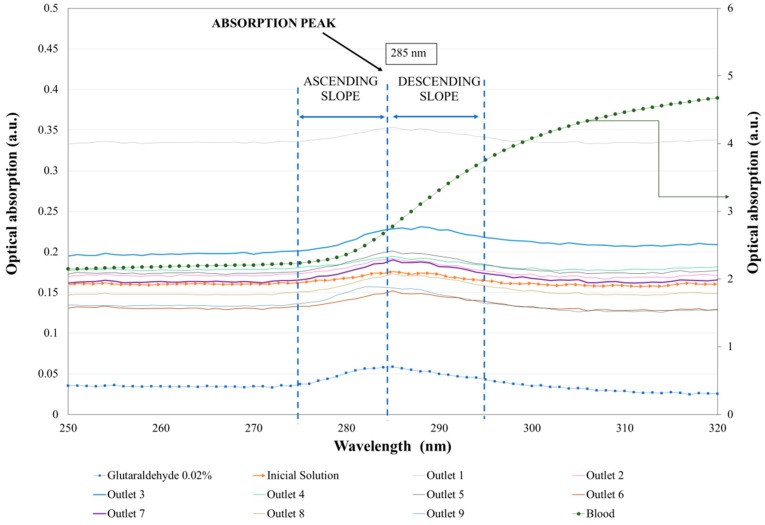
Average curves (n = 3) of the optical absorption spectra (a.u.) in the UV region of blood, glutaraldehyde, the initial working solution, and the samples collected in all the microchannel outlets (O1–O9). Note that the blood sample has an absolute optical absorption higher than all the other curves and, as a consequence, a secondary axis of optical absorption was added to the graphic (right axis).

With the aim of quantifying the separation process, Figure 4 presents the average values of the absorption spectra curves of the samples collected in the outlets (O1–O9) and their standard deviation. Each spectrum curve represents the average of three measurements. Additionally, the blood, glutaraldehyde, and initial solution spectra in the UV range (between 220 nm and 450 nm) are also presented. 

Firstly, the optical absorption of blood, glutaraldehyde and the initial solution (working fluid with both healthy and rigidified RBCs that enters the microfluidic device) in the UV region of the optical spectrum was measured. These measurements showed the different typical spectra of these solutions and function as a reference for the quantification of the separation efficiency. The main absorption peaks of the blood are located at 540 nm and 574 nm [33], both located in the visible region of the optical spectrum, and are therefore not shown in this Figure 4 plot. In the UV region, the blood (dashed green line) showed an absorption increase, with a wavelength of up to 320 nm. On the other hand, glutaraldehyde at 0.02% concentration (dashed blue line) had an absorption peak around 285 nm. Additionally, the initial solution (orange line with arrows in the plot) had, as expected, an intermediate shape, i.e., had the same spectrum peak as glutaraldehyde, but with a higher absorbance value (closer in intensity to the blood absorbance curve). This way, by quantifying the slopes in the glutaraldehyde peaks in all the solutions collected in the outlets, it was possible to quantify the relative proportion of rigidified RBCs and healthy RBCs in each region of the microfluidic device, helping to quantify the efficiency of the RBC separation. To clarify the values of optical absorption, Table 1 presents the average optical absorption values (a.u.) at the glutaraldehyde absorption peak (285 nm) with the respective standard deviation values.

Observing Figure 4, it is difficult to directly distinguish the optical absorption peaks of the samples collected in each outlet, making it hard to evaluate the proportion of healthy and chemically-modified RBCs in the microchannel outlets in order to evaluate the separation efficiency. However, from Figure 4 it is observed that while the blood optical absorption curve increased with the wavelength in the entire UV range, the glutaraldehyde sample presented an absorption peak at 285 nm. Therefore, by evaluating the absorption peak intensities of each sample, composed of RBCs with glutaraldehyde and healthy RBCs, it was possible to obtain the quantity of glutaraldehyde within the samples and, as a consequence, to evaluate the amount of RBCs chemically modified at each outlet. Using this strategy, it was possible to determine the ascending and descending slopes for each measured sample, using the expression slope = (y2 − y1)/(x2 − x1), where the x values correspond to the wavelength and the y values to the optical absorption. Table 2 presents the slopes calculated for two different regions, the ascending slope between 274 nm and 285 nm and descending slope between 285 nm and 296 nm. By evaluating the ascending slope region, it was observed that both glutaraldehyde and blood presented a positive slope, making it difficult to distinguish the effects of blood and glutaraldehyde in the obtained spectra. Therefore, we compared the slope in the descending region for all the samples (the slope in the blood sample was still positive, while in the presence of glutaraldehyde it became negative). Higher absolute values of this slope mean that the glutaraldehyde peak is more significant and, therefore, the sample has a larger quantity of modified RBCs and, consequently, is less deformable.

Due to the microchannel geometry, it was expected that the values at the outlets 1, 2, 3, and 4 would be symmetric to the values at the outlets 9, 8, 7, and 6, respectively. However, the results in Table 2 show slight differences, probably related to the stabilization of the flow inside the microchannel. Additionally, it was expected a decrease of RBCs with glutaraldehyde (and consequently a decrease in the absolute value of the descending slope) would correspond to the decrease in the gaps between pillars. For example, from outlet 1 to outlet 3, as the pillars gaps decreased from 17 µm to 14 µm, the quantity of modified RBCs in the outlet should have been lower, since for shorter distances between pillars only the highly deformable cells are able to cross these obstacles. Therefore, in O1 we should have had more glutaraldehyde than in O3, and in O9 we should have had more glutaraldehyde than in O7, in agreement with the results of Table 2. Furthermore, O5 should have had a higher value of glutaraldehyde-modified RBCs, since that outlet does not have any pillars working as obstacles and, therefore, less deformable RBCs are still able to reach that region. This is in agreement with the higher descending slope presented by the sample collected in this outlet. However, the results in Table 2 show some discrepancies from the expected results. O2 should have had a value between O1 and O3, and O8 should have had a value between O7 and O9. The discrepancies were likely caused by flow instabilities, some possible clogging problems due to the presence of stagnant flow regions, as well as some difficulties in microchannel fabrication, particularly when keeping equal distances between the pillars, which may have affected the separation results. These discrepancies were particularly common when comparing the left side of the microchannel (O9, O8, and O7) with the right side (O1, O2, and O3). Hence, further research on some critical parameters such as pillar spacing, layout, and orientation is needed. Nevertheless, the results obtained from this study are promising and are a starting point to develop an efficient microfluidic device based on cross-flow filtration for the partial separation of RBCs.

For the final intended application, concerning healthy and malaria-infected RBCs, additional tests must be performed in order to deal with real malaria parasite-affected RBC samples (at different disease stages) to compare disease and glutaraldehyde artificially impaired RBCs, establish target values, and fully validate this approach. Although it is well known that malaria leads to a decrease in RBC deformability, the target values are not yet clear, i.e., which deformability index values are achieved, since not many works can be found in the literature regarding this behavior (with testing in similar conditions). Barber et al. [36] is one of the few studies found by the authors showing experimental data regarding the deformation index of controls and malaria-infected RBCs. This study shows that, as expected, the malaria-infected cells are less deformable than the healthy cells (controls), and that RBC deformability is reduced in humans with malaria in proportion to the disease severity. However, absolute values cannot be compared, since the experimental work was performed under different stretching conditions. 

## 4. Limitations and Future Perspectives

Future work must focus on increasing the cell quantity in order to define the average property of the entire cell population with higher accuracy, since the physical properties of individual RBCs within the same RBC population can vary significantly. Additionally, since the ultimate goal is to develop an efficient separation device, more blood samples from different donors will be assessed to increase the RBCs’ variability and to include more independent data, as well as to increase the blood cells’ concentration in order to approximate to the physiological values. It will be also interesting to compare, for validation purposes, the quantification of the separation efficiency using deformability data for the RBCs measured within the microfluidic device outlets. For this purpose, the microdevice was already fabricated considering hyperbolic contractions before each outlet, since this is one of the most common methods to assess cells deformability [8,9,37]. Finally, it is intended to test real parasite-affected RBC samples to compare their behavior with the glutaraldehyde-modified RBCs [38], establish target values, and fully validate the proposed approach. This improved correlation will be used to relate the RBC behavior according to the various stages of malaria and to develop integrated sensors in microfluidic devices.

## 5. Conclusions

This study showed the potential of a cross-flow microfluidic device to perform partial passive separation of RBCs based on their deformability, as well as to assess RBC deformability by analyzing the acquired images and using spectrophotometry. Although the proposed microfluidic device requires further improvements, the results obtained from the present study suggest that devices based on cross-flow filtration are able to assure a simple, low cost, and efficient partial separation of RBCs in both physiological and pathological situations.

## Figures and Tables

**Table 1:** Average of the optical absorption values (a.u.) at the glutaraldehyde absorption peak (285 nm) obtained for each curve and respective standard deviations.

Samples	Average (n = 3)	Standard Deviations (n = 3)
**Glutaraldehyde**	0.059	±1.41 × 10^−8^
**Initial Solution**	0.175	±1.85 × 10^−8^
**O1**	0.353	±2.51 × 10^−6^
**O2**	0.192	±1.30 × 10^−8^
**O3**	0.228	±8.14 × 10^−9^
**O4**	0.195	±1.53 × 10^−8^
**O5**	0.201	±1.50 × 10^−8^
**O6**	0.152	±1.71 × 10^−8^
**O7**	0.190	±1.40 × 10^−8^
**O8**	0.173	±1.5 × 10^−8^
**O9**	0.156	±1.20 × 10^−8^

**Table 2:** Average ascending (274–285 nm) and descending slopes (285–296 nm) calculated for each sample.

Samples	Ascending Slope (274–285 nm)	Descending Slope (285–296 nm)
**Blood**	0.04979	0.09607
**Glutaraldehyde**	0.00217	−0.00165
**Initial Solution**	0.00130	−0.00109
**O1**	0.00165	−0.00129
**O2**	0.00172	−0.00149
**O3**	0.00249	−0.00105
**O4**	0.00138	−0.00111
**O5**	0.00237	−0.00171
**O6**	0.00182	−0.00141
**O7**	0.00233	−0.00168
**O8**	0.00214	−0.00155
**O9**	0.00183	−0.00177

## References

[1] Chen J., Chen D., Yuan T., Chen X., Xie Y., Fu H., Cui D., Fan X., Oo M.K.K. (2014). Blood plasma separation microfluidic chip with gradual filtration. Microelectron. Eng..

[2] Tanaka T., Ishikawa T., Numayama-Tsuruta K., Imai Y., Ueno H., Matsuki N., Yamaguchi T. (2012). Separation of cancer cells from a red blood cell suspension using inertial force. Lab Chip.

[3] Faustino V., Catarino S.S.O., Lima R., Minas G. (2016). Biomedical microfluidic devices by using low-cost fabrication techniques: A review. J. Biomech..

[4] Catarino S., Lima R., Minas G. (2016). Smart Devices: Lab-on-a-Chip.

[5] Tripathi S., Kumar Y.V.B.V., Prabhakar A., Joshi S.S., Agrawal A. (2015). Passive blood plasma separation at the microscale: A review of design principles and microdevices. J. Micromech. Microeng..

[6] Hou H.W., Bhagat A.A.S., Chong A.G.L., Mao P., Tan K.S.W., Han J., Lim C.T. (2010). Deformability based cell margination—A simple microfluidic design for malaria-infected erythrocyte separation. Lab Chip.

[7] Guo Q., Duffy S.P., Matthews K., Deng X., Santoso A.T., Islamzada E., Ma H. (2016). Deformability based sorting of red blood cells improves diagnostic sensitivity for malaria caused by Plasmodium falciparum. Lab Chip.

[8] Faustino V., Pinho D., Yaginuma T., Calhelha R.C., Ferreira I.C., Lima R. (2014). Extensional flow-based microfluidic device: Deformability assessment of red blood cells in contact with tumor cells. BioChip J..

[9] Bento D., Rodrigues R.O., Faustino V., Pinho D., Fernandes C.S., Pereira A.I., Garcia V., Miranda J.M., Lima R. (2018). Deformation of red blood cells, air bubbles, and droplets in microfluidic devices: Flow visualizations and measurements. Micromachines.

[10] Li H., Papageorgiou P.D., Chang H.-Y., Lu L., Yang J., Deng Y. (2018). Synergistic integration of laboratory and numerical approaches in studies of the biomechanics of diseased red blood cells. Biosensors.

[11] Tomaiuolo G. (2014). Biomechanical properties of red blood cells in health and disease towards microfluidics. Biomicrofluidics.

[12] Xue C., Wang J., Zhao Y., Chen D., Yue W., Chen J. (2015). Constriction Channel Based Single-Cell Mechanical Property Characterization. Micromachines.

[13] Suresh S., Spatz J., Mills J.P., Micoulet A., Dao M., Lim C.T., Beil M., Seufferlein T. (2005). Connections between single-cell biomechanics and human disease states: Gastrointestinal cancer and malaria. Acta Biomater..

[14] Agrawal R., Smart T., Nobre-Cardoso J., Richards C., Bhatnagar R., Tufail A., Shima D., Jones P.H., Pavesio C. (2016). Assessment of red blood cell deformability in type 2 diabetes mellitus and diabetic retinopathy by dual optical tweezers stretching technique. Sci. Rep..

[15] Boas L., Faustino V., Lima R., Miranda J., Minas G., Fernandes C., Catarino S. (2018). Assessment of the Deformability and Velocity of Healthy and Artificially Impaired Red Blood Cells in Narrow Polydimethylsiloxane (PDMS) Microchannels. Micromachines.

[16] Chen X., Cui D., Liu C., Li H. (2008). Microfluidic chip for blood cell separation and collection based on crossflow filtration. Sens. Actuators B Chem..

[17] Gidaspow D., Huang J. (2009). Kinetic Theory Based Model for Blood Flow and its Viscosity. Ann. Biomed. Eng..

[18] Sankar D.S., Nagar A.K., Kumar A.V. (2015). Mathematical analysis of single and two-phase flow of blood in narrow arteries with multiple contrictions. J. Appl. Fluid Mech..

[19] Saha R.K., Karmakar S., Roy M. (2012). Computational Investigation on the Photoacoustics of Malaria Infected Red Blood Cells. PLoS ONE.

[20] Diez-Silva M., Dao M., Han J., Lim C.-T., Suresh S. (2010). Shape and Biomechanical Characteristics of Human Red Blood Cells in Health and Disease. MRS Bull..

[21] Handayani S., Chiu D.T., Tjitra E., Kuo J.S., Lampah D., Kenangalem E., Renia L., Snounou G., Price R.N., Anstey N.M. (2009). High deformability of Plasmodium vivax-infected red blood cells under microfluidic conditions. J. Infect. Dis..

[22] Shelby J.P., White J., Ganesan K., Rathod P.K., Chiu D.T. (2003). A microfluidic model for single-cell capillary obstruction by Plasmodium falciparum-infected erythrocytes. Proc. Natl. Acad. Sci. USA.

[23] Garcia V., Dias R., Lim R. (2012). In vitro blood flow behaviour in microchannels with simple and complex geometries. Applied Biological Engineering—Principles and Practice.

[24] Guillou L., Dahl J.B., Lin J.-M.G., Barakat A.I., Husson J., Muller S.J., Kumar S. (2016). Measuring Cell Viscoelastic Properties Using a Microfluidic Extensional Flow Device. Biophys. J..

[25] Pinho D., Yaginuma T., Lima R. (2013). A microfluidic device for partial cell separation and deformability assessment. BioChip J..

[26] Gossett D.R., Tse H.T.K., Lee S.A., Ying Y., Lindgren A.G., Yang O.O., Rao J., Clark A.T., Di Carlo D. (2012). Hydrodynamic stretching of single cells for large population mechanical phenotyping. Proc. Natl. Acad. Sci. USA.

[27] Henon Y., Sheard G.J., Fouras A. (2014). Erythrocyte deformation in a microfluidic cross-slot channel. RSC Adv..

[28] Pinho D., Campo-Deaño L., Lima R., Pinho F.T. (2017). In vitro particulate analogue fluids for experimental studies of rheological and hemorheological behavior of glucose-rich RBC suspensions. Biomicrofluidics.

[29] Lee S.S., Yim Y., Ahn K.H., Lee S.J. (2009). Extensional flow-based assessment of red blood cell deformability using hyperbolic converging microchannel. Biomed. Microdevices.

[30] Yaginuma T., Oliveira M.S.N., Lima R., Ishikawa T., Yamaguchi T. (2013). Human red blood cell behavior under homogeneous extensional flow in a hyperbolic-shaped microchannel. Biomicrofluidics.

[31] Rodrigues R.O., Pinho D., Faustino V., Lima R. (2015). A simple microfluidic device for the deformability assessment of blood cells in a continuous flow. Biomed. Microdevices.

[32] Rodrigues R.O., Bañobre-López M., Gallo J., Tavares P.B., Silva A.M.T., Lima R., Gomes H.T. (2016). Haemocompatibility of iron oxide nanoparticles synthesized for theranostic applications: A high-sensitivity microfluidic tool. J. Nanopart. Res..

[33] Silva I., Lima R., Minas G., Catarino S.O. (2017). Hemozoin and Hemoglobin Characterization by Optical Absorption Towards a Miniaturized Spectrophotometric Malaria Diagnostic System. Proceedings of the 5th IEEE Portuguese BioEngineering Meeting.

[34] Pinto V.C., Sousa P.J., Cardoso V.F., Minas G. (2014). Optimized SU-8 processing for low-cost microstructures fabrication without cleanroom facilities. Micromachines.

[35] Rodrigues R.O., Lopes R., Pinho D., Pereira A.I., Garcia V., Gassmann S., Sousa P.C., Lima R. (2016). In vitro Blood Flow and Cell-Free Layer in Hyperbolic Microchannels: Visualizations and Measurements. BioChip J..

[36] Barber B.E., Russell B., Grigg M.J., Zhang R., William T., Amir A., Lau Y.L., Chatfield M.D., Dondorp A.M., Anstey N.M. (2018). Reduced red blood cell deformability in Plasmodium knowlesi malaria. Blood Adv..

[37] Raquel R., Vera F., Elmano P., Diana P., Rui L. (2013). Red Blood Cells deformability index assessment in a hyperbolic microchannel: The diamide and glutaraldehyde effect. WebmedCentral. Biomed. Eng..

[38] Hosseini S.M., Feng J.J. (2012). How malaria parasites reduce the deformability of infected red blood cells. Biophys. J..

